# Role of Oral Retinoids in Treatment of Acne Vulgaris With a Bioinformatics-Based Perspective of Personalized Medicine

**DOI:** 10.7759/cureus.38019

**Published:** 2023-04-23

**Authors:** Renee Scott-Emuakpor, Kiranmayi Vuthaluru, Abhijit Nagre, Inshal Jawed, Priyansh A Patel, Harmandeep K Sidhu

**Affiliations:** 1 Dermatology, International European University, Kyiv, UKR; 2 Pediatrics, Jawaharlal Nehru Medical College, Belgaum, IND; 3 Medicine, Topiwala National Medical College & Bai Yamunabai Laxman (BYL) Nair Charitable Hospital, Mumbai, IND; 4 Internal Medicine, Dow Medical College, Karachi, PAK; 5 Medicine, Byramjee Jeejeebhoy (BJ) Medical College, Ahmedabad, IND; 6 Medicine, Medical College Baroda, Baroda, IND; 7 Dermatology, Dayanand Medical College and Hospital, Ludhiana, IND; 8 Medicine, Indira Gandhi Government Medical College and Hospital, Nagpur, IND

**Keywords:** gene, tgfβ, skin dryness, propionibacterium, oral retinoid, acne vulgaris

## Abstract

Acne vulgaris is a skin condition characterized by the inflammation or hyperactivity of sebaceous glands on the skin, which results in the creation of comedones, lesions, nodules, and perifollicular hyperkeratinization. Increased sebum production, follicular blockage, and bacterial colonization may contribute to the disease etiology. Environmental factors, hormonal imbalance, and genetic predisposition can alter the severity of the disease. Its mental and monetary effects can be problematic for the society. In this study, we examined the role of isotretinoin in the treatment of acne vulgaris based on evidence from prior research. This review literature study compiled publications on the treatment of acne vulgaris from 1985 to 2022 based on PubMed and Google Scholar publications. Additional bioinformatics analyses were accompanied by GeneCards, STRING model, and DrugBank databases. These complementary analyses were designed to obtain a better perspective of personalized medicine which is highly required for dose-precise administrations of acne vulgaris treatment. Isotretinoin has been recognized as an effective treatment for acne vulgaris, particularly in cases that have been resistant to previous medications or have resulted in scarring, according to gathered data. Oral isotretinoin inhibits the proliferation of Propionibacterium acne, a critical factor in the development of acne lesions; also, it has been shown to be effective in reducing the number of Propionibacterium-resistant patients and regulating sebum production and reducing sebaceous gland size more effectively than other treatment options resulting in general improvements in skin clarity and acne severity and reduce inflammatory in 90% of patients. In addition to its efficacy, the majority of patients have shown that oral isotretinoin is well tolerated. This review highlights the use of oral retinoids, particularly isotretinoin, as an effective and well-tolerated treatment option for acne vulgaris. It has been proven that oral isotretinoin is useful for achieving long-lasting remission in patients with severe or resistant instances. Despite the fact that oral isotretinoin is related to a number of potential harmful effects, skin dryness was the most common side effect reported by patients that can be managed with the aid of suitable monitoring and drug administration against specific genes identified by genotyping of the susceptible variants of genes involved in TGFβ signaling pathway.

## Introduction and background

Acne vulgaris is a chronic condition characterized by the obstruction and irritation of pilosebaceous glands. Acne can manifest as non-inflammatory lesions, inflammatory lesions, or a combination of both, affecting the face, back, and chest primarily [1-3]. Severe acne is typically characterized by a large number of lesions, including nodules and cysts, and causes permanent disfigurement and scarring, resulting in low self-esteem and psychological distress that negatively impacts the quality of life [4-6]. Previous epidemiology data reported that acne vulgaris affects approximately 250 adolescents aged 12 to 17 per thousand [7]. Between 80 and 95% of persons are affected by clinical acne vulgaris at some point before the age of 30 [8]. In the past decade, according to the Global Burden of Disease Study 2010 with an estimated worldwide incidence (for all ages) of 9.38%, acne vulgaris (henceforth referred to as acne) is the eighth most prevalent skin disease [9]. Acne prevalence data ranges by country and age group, with estimates ranging from 35% to near to 100% of teenagers suffering from acne at some stage [10].

Acne is moderate to severe in 15-20% of teenagers, which includes the majority of teens from 15-17 years [11-15]. According to prevalence rates of acne by age and statistics from the 1996 Census, 40-50 million people in the United States have acne, with an incidence rate of 85% among those between the ages of 12 and 24 [16, 17]. According to a research by Lucky et al., 50% of boys between the ages of 10 and 11 had more than 10 comedones, and the severity of acne in males was found to be connected with pubertal maturation [18]. The same team's research found that 78% of girls between the ages of 8 and 12 have acne [19]. Importantly, prepubertal girls having severe acne had significantly greater dehydroepiandrosterone sulphate levels than girls with milder acne [18, 19]. According to extensive research conducted in the UK, France, and the USA, acne regularly ranks among the top three skin problems that are most common in the general population [20-22]. Early adolescence is when acne first manifests itself as face sebum production, comedones, and inflammatory lesions [14, 23]. Prepubescent children can develop acne, although this is often non-inflammatory in character since children have not yet started producing sebum, which creates the ideal environment for P. acnes to thrive [24]. The majority of acneiform lesions, which occur in 20% of neonates, fade away by three months [25]. According to a Danish research, males' average onset of puberty has decreased during a 15-year period from a mean age of 11.92 years to 11.66 years [26]. Younger patients between the ages of 8 and 11 were coming with acne in their clinics led Friedlander et al. to hypothesize that this earlier age of puberty was the cause [24]. Acne is a persistent condition that, for unknown reasons, can occasionally last into adulthood [27-30]. According to a survey of the German population, 64% of people between the ages of 20 and 29 and 43% of people between the ages of 30 and 39 had visible acne, while 3% of men and 5% of women between the ages of 40 and 49 continued to have acne [31, 32]. Cohort studies which follow the progression of acne through time in the same people are more reliable than sequential prevalence studies for demonstrating that acne declines with age. Studies on self-reported acne seem to be often unverified [10].

Acne's pathogenesis is intricate and complicated. The etiopathogenesis of acne is recognized to involve four components: increased sebum secretion, aberrant follicular keratinization, bacterial proliferation, and inflammation. Additionally, food, genetic factors, and oxidative stress might affect the progression of acne [33-36]. The components of acne's pathogenicity are agreed upon by clinicians, however, the sequence of events leading to its development is contested. In the past, it was believed that aberrant keratinization led to the formation of acne's earliest subclinical manifestation, microcomedones [37, 38]. Next, inflammation with androgens that act on sebaceous glands, excessive sebum production, keratin plug development, P. acnes colonization, and innate immunity activation occurred [39-41]. However, the involvement of the commensal bacterium Propionibacterium acne in the inflammatory phase of acne remains debatable [41-43]. Controlling the four pathogenic pathways involved in the development of acne lesions is essential for therapeutic success [44]. Antibiotics such as clindamycin and doxycycline; anti-keratolytic such as benzoyl peroxide and salicylic acid; and Vitamin A analogues such as retinoic acids and isotretinoin are among the various acne treatments available that address distinct underlying causes. These remedies are further classified as Topical and Oral. Acne treatment can be either monotherapy or polytherapy [44, 45]. Oral retinoids are a very efficient treatment for acne vulgaris. They function by inhibiting the formation of keratin, a protein that can clog pores and cause acne [45]. Oral retinoids are typically reserved for severe cases of acne that do not respond to topical creams and gels. Isotretinoin, acitretin, and tretinoin are three well-known oral retinoids used to treat acne. Moderate to severe acne is treated with systemic therapy such as oral antibiotics, oral retinoids such as isotretinoin, or hormone treatment [46-49].

Even after significant researches, it remains uncertain whether oral retinoids are the most effective treatment for acne vulgaris. The current paper assessed the efficacy of the various oral retinoids regimens for the treatment of acne vulgaris.

## Review

Methods

From 1985 until 2022, this literature study compiled publications on the treatment of acne vulgaris. There have been 2,108 publications indexed in PubMed and 8,630 citations in Google Scholar containing Acne Vulgaris in their titles. This extensive time frame provides strong evidence supporting the importance of oral isotretinoin in the treatment of acne vulgaris (179 results from PubMed), while allowing for the time required for the development of novel treatment strategies over time (23 results). Overall, all articles suggest that oral isotretinoin is significantly effective in the treatment of acne vulgaris. Further bioinformatics analyses were conducted according to a previously published Genome-Wide Association Study (GWAS) and performed by GeneCards (https://www.genecards.org/), STRING model (https://string-db.org/), and DrugBank (https://go.drugbank.com/) databases. These complementary assessment designs are based on the perspective of personalised medicine-based diagnosis which is highly required for dose-precise administrations of acne vulgaris treatment as narrow as it can be possible.

Result

As first-line treatments for mild acne vulgaris, benzoyl peroxide and topical retinoids were not as successful for treating severe acne. In 1982, oral isotretinoin was launched and became the treatment of choice for severe acne [50]. Several investigations have shown that the mechanism of action of oral isotretinoin involves inhibiting sebum production and reducing sebum gland size, as well as modifying vitamin A metabolism, and that oral isotretinoin has an effective mechanism for treating acne vulgaris [51, 52]. In addition, oral isotretinoin has been demonstrated to be more successful than oral antibiotics in producing persistent remission after treatment [47, 53-55]. According to the American Academy of Dermatology's "Treatment Guidelines," some of the indications for oral isotretinoin include severe nodular acne, moderate acne that has failed therapy, and acne with scarring [56].

A research was conducted to assess the efficacy and safety of isotretinoin in comparison to a similar reference product. The researchers conducted a bicentric study with participants aged 13 to 35 who had moderate to severe acne. The starting dose of isotretinoin ranged from 0.5 mg/kg to 120 mg/kg each day. By the final visit after eight months, the number of counted lesions had decreased by 99%. At the conclusion of the study, 42.6% of subjects suffered erythema, 53.2% observed skin peeling, and 48.9% complained of dry skin. Nonetheless, it should be noted that no major adverse effects were recorded during this trial. Picosse et al. found that oral isotretinoin was just as effective, safe, and well tolerated as comparable reference medicines, and provided a considerable improvement in the participants' overall quality of life. In order to lessen the psychological cost of post-acne vulgaris, this study advises oral isotretinoin early medical intervention to diminish follicular inflammation and minimize the appearance of scars [57]. In the study "Consensus on the Use of Oral Isotretinoin in Dermatology" conducted by the Brazilian Society of Dermatology, reviewers unanimously agreed on the efficacy and application of oral isotretinoin in the treatment of acne vulgaris and post-inflammatory scarring. Dryness around the mucosal regions of the face was the most common side effect (Bagatin et al., 2020). In addition, retardation, depression, and inflammatory bowel disease were exhaustively evaluated for adverse effects, and it was concluded that there was no association between oral isotretinoin use and the aforementioned conditions [58].

Different dose regimens of oral isotretinoin for acne vulgaris were the subject of a randomized, controlled trial by Agarwal et al. [59]. Participants having a history of mild, moderate, and severe acne were separated into four unique categories over the course of 16 weeks. Patients in Group A received 1 mg/kg of oral isotretinoin daily, whereas patients in Group B received 1 mg/kg of oral isotretinoin every other day. In contrast, participants in Group C were provided 1 mg/kg of oral isotretinoin each day throughout weeks one and four. Group D patients were provided oral isotretinoin at 20 mg/kg every other day for 16 weeks. In comparison to their counterparts in other groups, patients with severe acne who had a high-dose treatment progressed the most and the quickest, as demonstrated by the results. In addition, people who classified their acne as minor demonstrated comparable findings across cohorts. However, those with moderate acne responded effectively to a large oral dose of isotretinoin. Therefore, the group that received a higher dose of isotretinoin experienced a bigger reduction in inflammatory lesions, acne, and nodules than the group that took the medication for a shorter duration. Over time, the patients demonstrated considerable happiness and alleviation. Lastly, reports were made of treatment-related adverse effects including dry skin. Similarly, dry skin was the most prevalent adverse impact reported in other reviewed publications [59].

**Figure 1:**
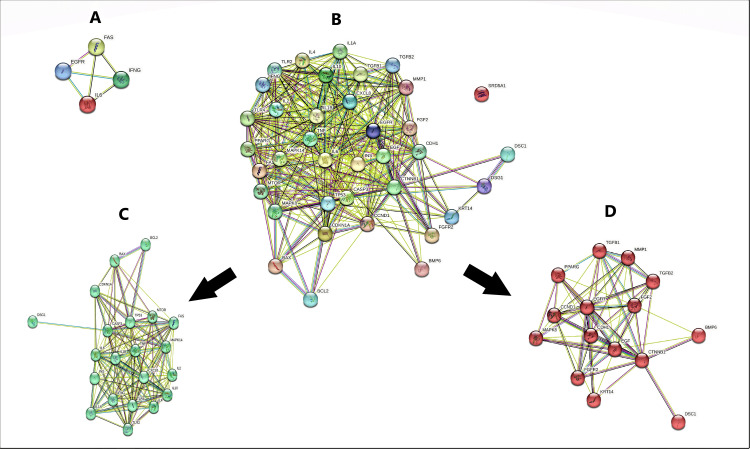
The modeled networks via STRING MODEL. A) The four genes with high overlapping score involved in all three roots (Acne vulgaris, TGFβ signaling pathway, and isotretinoin). B) All 37 genes with overlapping role at least in two roots (Major network). C) Cluster I containing IL6, FAS, and IFNG gene (minor network). D) Cluster II containing EGFR gene.

Considering the dose-dependency of oral isotretinoin treatment, the genetic affinity of a person to receive different dosage must be investigated. To the best of our knowledge, there are limited studies focusing on this subject. Large families and twin studies are providing more and more evidence that genetic variables play a role in the development of acne [60, 61]. Previous studies reported a number of potential genes including tumor necrosis factor (TNF), tumor necrosis factor receptor 2 (TNFR2), toll-like receptor 2 (TLR2), interleukin-1 alpha (IL-1), cytochrome P450 family 1 subfamily A polypeptide 1 (CYP1A1), cytochrome P450 family 17 subfamily A polypeptide 1 (CYP17A1), cytochrome P450 family 21 subfamily A poly (AR) [62-69]. Two important cellular processes are impacted by these genes: the modulation of steroid hormone metabolism and the innate immunological responses of epidermal keratinocytes [70]. According to Navarini et al., they performed a genome-wide association analysis of loci with acne; they are 11q13.1 containing rs478304, 5q11.2 containing rs38055, and 1q41 containing rs1159268. All three loci have genes including OVOL1, FST and TGFB2 genes associated with the TGFβ cell signaling pathway. They added that OVOL1 and TFGB2 transcripts have decreased expression in affected compared with normal skin. As a final result in accordance with susceptibility to acne, their findings introduced a vital role for dysregulation of TGFβ-mediated signaling pathway [71]. Another genome-wide association analysis conducted by He et al. introduced two susceptible loci at 11p11.2 containing rs747650 and rs1060573 and 1q24.2 containing rs7531806. Both loci include genes DBB2 and SELL, which have functions in the metabolism of androgen, inflammation mechanism and formation of scar in severe acne [70]. The strategy of the present review was to design a network based on three roots including genes involved in TGFβ signaling pathway, genes related to acne vulgaris pathogenicity, and genes associated with isotretinoin. The mentioned genes were obtained from 100 highly scored genes in GeneCards, then by filtering the overlapping genes (at least with two roots), four genes were involved in all three roots including IL6, EGFR, FAS, and IFNG. The string model indicated that all of these four genes are linked with each other (Figure 1B). In the next step, duplicated genes were discarded and 37 genes were remained. The STRING model of these genes was drawn (major network) and then, by clustering the aforementioned genes, two clusters were drawn (minor networks) (Figure 1C and Figure 1D). On further evaluation it was found that IL6, FAS, and IFNG were more associated than EGFR hence more concentration was put on the former three genes. Further investigations by DrugBank indicated that Foreskin keratinocyte commonly targets FAS and INFG proteins.

Discussion

Acne vulgaris is a prevalent skin ailment that affects millions of people worldwide and can have substantial medical and psychological consequences for those affected. Therefore, it is essential to have a variety of appropriate therapy alternatives for this ailment. Oral retinoids, particularly isotretinoin, have been demonstrated to be an effective treatment for acne vulgaris [72]. According to data collected between 1985 and 2022, isotretinoin has been identified as an effective treatment for acne vulgaris, especially in cases that have been resistant to prior therapies or have resulted in scarring [53, 54]. It is believed that the mechanism of action of oral isotretinoin in the treatment of acne vulgaris involves alterations in vitamin A metabolism, suppression of sebum production, and restriction of sebaceous gland growth [51, 52]. These processes limit the proliferation of Propionibacterium acnes, a critical factor in the development of acne lesions, and can lead to improvements in skin clarity and acne severity overall. In 90% of patients, the inflammatory lesions were observed to be decreased [57]. Oral isotretinoin is more successful than oral antibiotics in attaining durable remission after therapy, according to an assessment of the relevant research. This is significant because it shows that oral isotretinoin may be a more permanent treatment option for acne, perhaps lowering the need for numerous rounds of treatment and the expenses and burdens associated with them. Several studies have demonstrated its efficacy in reducing the number of Propionibacterium-resistant patients and its superior capacity to regulate sebum production and reduce sebaceous gland size in comparison to other treatment alternatives [50, 53, 54]. This research consequently supports the use of oral isotretinoin as the therapy of choice for severe acne.

In addition to its efficacy, oral isotretinoin has been demonstrated to be well tolerated by the majority of patients. Although it is associated with a number of potential adverse effects, the reviewed literature suggests that the majority of these side effects are mild and temporary, and may be handled with appropriate monitoring and management measures. The most prevalent adverse effect reported by patients was skin dryness. Given that isotretinoin inhibits hyperactive seborrheic glands, this may result in dryness and flaking. In addition to isotretinoin treatment, it is vital to recommend proper hydration, sun protection, and moisturization. This is in contrast to conventional acne treatments, such as oral antibiotics, which can cause a variety of adverse effects and may not be as helpful in enhancing patients' quality of life [58, 59]. Numerous studies suggest that oral isotretinoin significantly reduces the number of acne lesions and improves overall skin clarity. Particularly, oral isotretinoin looks more effective at achieving prolonged remission after treatment than other choices such as oral antibiotics, making it a potentially more durable option for acne patients. This study has significant implications for the current and future management of patients with hormonal or environmental acne [57, 59, 73].

The results of analyses based on a GWAS study revealed that the overlapping genes involved in three roots including acne vulgaris pathology pathway, TGFβ signaling pathway, and isotretinoin action pathway (300 genes in total) are 36 genes, among them, IL6, FAS, and IFNG genes were the most potential candidates because of their overlapping link in all three mentioned roots. Further investigations uncovered foreskin keratinocyte as a common FDA-approved drug basis which might be a new introduction as the second line of acne vulgaris treatment. Keratinocytes establish the main barrier and warn the host of impending danger by secreting a variety of cytokines and chemokines including interleukin [IL]1α, IL6, tumor necrosis factor (TNF), and prostaglandins [74-78]. It has been demonstrated that P. acnes can activate TLR2 and that P. acnes induces the secretion of IL12 and IL8 by primary human monocytes and IL6 by macrophages through this cell surface receptor [79]. TGF's biology supports a pathogenesis-related involvement in acne. TGF prevents keratinocyte hyperproliferation that can result in comedo development and follicular blockage in acne [80-83]. Similarly, sebaceous gland lipid production - which is elevated and changed in acne - is decreased by TGF. The innate immune responses that P. acnes elicits during microbial colonization might be additionally regulated by TGF [79, 84-88].

Foreskin keratinocytes are skin cells that are cultivated as a skin cell substitution for wounds in order to speed up wound closure and recovery [89]. In 1975, Rheinwald and Green succeeded in growing human keratinocytes on lethally irradiated mouse fibroblasts. For the first time, O'Conner and his colleagues used cultured autologous epithelium to cover burn defects in 1981. Using mesenchymal cells like fibroblasts, a dermal replacement based on collagen I gel was built to provide a "living" alternative. This method was described as "skin equivalent," "composite culture," or "organotypical culture" when an epidermal layer was added [90]. Several skin replacements used for different causes contain foreskin keratinocytes as a key component [89]. Neonatal foreskin fibroblast and keratinocytes are combined to produce the medication Apligraf (Organogenesis, Canton, MA), which is made from keratinocytes obtained from neonatal foreskins. The matrix for cell growth and differentiation is a bovine collagen gel. Apligraf has been shown to be effective in the treatment of venous leg ulcers and diabetic foot ulcers by boosting wound healing rates and lowering the time necessary for wound closure [91-95]. OrCel (Forticell Bioscience, Englewood Cliffs, NJ), another skin alternative, is similar to Apligraf in that it comprises both fibroblasts and keratinocytes produced from the neonatal foreskin, but it also uses a type I collagen sponge as its matrix. It is grafted onto partial-thickness wounds, where it provides a suitable matrix for host cell migration [96, 97].

When interpreting the conclusions of this literature review, a few limitations must be considered. The review only includes articles published between 1985 and 2022, which may not represent the full scope of research on the topic. It is likely that additional information regarding the efficacy and tolerability of oral isotretinoin for the treatment of acne vulgaris could be gleaned from more recent research not included in this analysis. The evaluation includes research with a variety of sample sizes and study methodologies, which may have affected the generalizability of the findings. In spite of these limitations, this literature evaluation supports the use of oral isotretinoin as a first-line treatment for moderate to severe acne vulgaris. Future studies should continue to investigate the long-term efficacy and safety of oral isotretinoin, as well as techniques to decrease the risk of adverse effects and optimize treatment outcomes for acne vulgaris patients.

## Conclusions

This review supports the use of oral retinoids, specifically isotretinoin, as an effective and well-tolerated acne vulgaris therapy option. It has been demonstrated that oral isotretinoin is beneficial in attaining lasting remission following treatment of severe or resistant cases of the illness. Patients with acne vulgaris have a valuable therapy option with oral retinoids, despite their limitations, which include the possibility of adverse effects and the need for thorough monitoring and control. Interestingly, by bioinformatics analysis, personalized medicine-based diagnosis in combination with the oral isotretinoin is suggested to improve the efficacy of dose-dependency administrations. This review highly recommended further association studies for the candidate genes including IL6, FAS, and IFNG to identify the genetic variants playing unknown roles in the pathogenicity of acne vulgaris and receiving medications.
